# Effect of high hydrostatic pressure on *in vitro* digestibility and techno-functional properties of fava bean (*Vicia faba* L.) protein

**DOI:** 10.3389/fnut.2026.1862046

**Published:** 2026-07-09

**Authors:** Mauricio Opazo-Navarrete, Cristina Bravo-Reyes, César Burgos-Díaz, Manuel Chacón-Fuentes, Dominique Larrea-Wachtendorff, Rubén Agregán, Rubén Domínguez-Valencia, José Manuel Lorenzo

**Affiliations:** 1 Agriaquaculture Nutritional Genomic Center (CGNA), Temuco, Chile; 2 Núcleo de Investigación en Producción Alimentaria, Facultad de Recursos Naturales, Universidad Católica de Temuco, Temuco, Chile; 3 FoodTech SpA, Temuco, Chile; 4 Dirección de Innovación y Transferencia Tecnológica, Vicerrectoría de Investigación y Posgrado, Universidad Católica de Temuco, Temuco, Chile; 5 Department of Food Engineering, Universidad del Bío-Bío, Chillán, Chile; 6 Centro Tecnolóxico da Carne de Galicia, Parque Tecnolóxico de Galicia, San Cibrao das Viñas, Spain; 7 Área de Tecnoloxía dos Alimentos, Facultade de Ciencias, Universidade de Vigo, Ourense, Spain

**Keywords:** fava bean protein, functional properties, high hydrostatic pressure, *in vitro* digestion, protein secondary structure

## Abstract

The increasing demand for sustainable protein sources has positioned fava beans as a promising alternative; however, their limited techno-functional properties and digestibility restrict broader application within food systems. This study aimed to evaluate the impact of high hydrostatic pressure on the structural, nutritional, and techno-functional properties of fava bean protein isolate under various pressure levels, treatment times, and protein concentrations. Protein dispersions (5–20% w/v) were subjected to pressures ranging from 300 to 600 MPa for up to 9 min, and subsequent changes in protein structure, *in vitro* digestibility, and functional properties were assessed. Moderate pressures (300–400 MPa) induced partial protein unfolding, thereby increasing the exposure of reactive groups and enhancing enzymatic accessibility, which resulted in improved digestibility, solubility, emulsifying capacity, foaming properties, and water holding capacity. In contrast, higher pressures (≥500 MPa) and extended treatment times promoted protein aggregation, leading to reduced functionality and digestibility. Furthermore, elevated protein concentrations limited structural modifications due to molecular steric hindrance, thereby reducing responsiveness to pressure treatment. These findings demonstrate a biphasic, pressure-dependent behavior whereby controlled structural disruption enhances protein performance, whereas excessive processing induces aggregation and functional decline. Overall, moderate HHP conditions (300–400 MPa) were identified as an effective strategy to enhance the nutritional and techno-functional quality of fava bean protein, offering valuable insights for the development of sustainable plant-based food ingredients.

## 1 Introduction

The global demand for plant-based proteins is rapidly increasing, driven by a growing population and heightened concerns over the environmental sustainability and ecological footprint of animal protein production (1, 2). Fava bean (*Vicia faba* L.), also known as broad or horse bean, has emerged as an exceptional candidate to meet this demand due to its high protein content (typically ranging from 20% to 41%), easy cultivation, and excellent nitrogen-fixing capacity, which improves soil fertility (3, 4). In addition to its macromolecular components, fava beans are rich in dietary fiber, minerals, and bioactive compounds with potential health benefits, including antioxidant, antihypertensive, and anti-inflammatory properties (5–7).

Nevertheless, the utilization of fava bean protein within the food industry remains relatively limited in comparison to animal-derived proteins, such as those obtained from milk or eggs, primarily attributable to its poor solubility and limited techno-functional properties (8). Moreover, similar to other legumes, fava beans contain various antinutritional factors, including tannins, phytic acid, protease inhibitors, and the pyrimidine glucosides vicine and convicine, which can impede protein digestibility and mineral bioavailability (9, 10). Conventional thermal treatments are frequently employed to enhance these attributes by deactivating heat-sensitive antinutrients; nonetheless, excessive heating may result in protein denaturation, undesirable “cooked” flavors, and the degradation of beneficial bioactive compounds (11, 12).

High Hydrostatic Pressure (HHP) has emerged as an effective non-thermal method for food preservation and the modification of macromolecules (13, 14). Typically operating within a range of 100 to 800 MPa, HHP utilizes the isostatic principle, whereby pressure is transmitted instantaneously and uniformly throughout the food matrix, regardless of its dimensions or shape (15, 16). In accordance with Le Chatelier's principle, HHP induces shifts in molecular structures toward states of reduced volume, thereby disrupting non-covalent interactions such as hydrophobic and electrostatic bonds, while preserving primary covalent bonds (17, 18). These structural changes, including protein unfolding and the dissociation of oligomeric assemblies into monomers, can be strategically employed to modify the techno-functional properties of plant proteins (19, 20).

Recent research has indicated that HHP can improve the functional properties of various legume proteins, including their solubility, emulsifying properties, and foaming capacity (21–25). Moreover, the structural loosening and unfolding of proteins during HHP can expose previously buried enzymatic cleavage sites, potentially enhancing *in vitro* digestibility and facilitating the release of bioactive peptides (26). While some studies have explored the effects of HHP on the structure and function of other pulses, a systematic investigation into how different pressure levels, treatment times, and protein concentrations impact the techno-functional properties and nutritional quality of fava bean protein remains relatively limited (14, 27).

Therefore, the objective of this study was to evaluate the effect of HHP on the *in vitro* digestibility and techno-functional properties, specifically solubility, emulsification, foaming, and gelation, of fava bean protein. By exploring various pressure-time combinations, this research seeks to optimize HHP parameters to enhance the utilization of fava bean protein as a functional and nutritious ingredient in the development of sustainable, plant-based food products.

## 2 Materials and methods

### 2.1 Materials

Fava bean protein isolate (FBPI) was purchased from Prinal SA (Santiago, Chile). All chemicals and reagents utilized in this study were of analytical grade and obtained from Sigma-Aldrich (St. Louis, MO, USA).

### 2.2 Chemical analysis of FBPI

The nitrogen content was quantified utilizing the Dumas method with the DUMATHERM^®^ N PRO analyzer (Königswinter, Germany), employing a conversion factor of 6.25. Lipid content was determined according to AOAC official method 945.18. Moisture content was determined following NCh 841 Of.78. Total dietary fiber was measured utilizing AOAC official method 991.43. Ash content was established according to AOAC official method 942.05. Carbohydrate content was calculated by difference as nitrogen-free extracts.

### 2.3 Amino acid analysis of FBPI

The amino acid (AA) profile of the FBPI samples was assessed utilizing standardized analytical procedures. Tryptophan and methionine were quantified in accordance with EU method 152/2009, while cysteine was analyzed using an oxidative protocol based on ISO 13903:2005. The remaining amino acids were quantified subsequent to acid hydrolysis following ISO 13903:2005 guidelines.

### 2.4 Material preparation

FBPI suspensions were prepared by diluting FBPI in purified water at various concentrations (5%, 10%, 15%, and 20%), representing the range commonly used in plant protein ingredient formulations and food applications, from dilute dispersions to concentrated systems where protein-protein interactions become increasingly relevant. The pH of each solution was adjusted to 7.0 using 1 M NaOH. The mixtures were stirred for 1 h at 5,000 rpm employing an Ultraturrax (IKA, Germany), and subsequently transferred into 250 mL PET bottles. The bottles were allowed to hydrate overnight at ambient temperature prior to pressurization.

### 2.5 High hydrostatic pressure (HHP) treatment

FBPI dissolutions were packaged in sealed containers and subjected to HHP at ambient temperature (20 ± 1 °C) using an isostatic processing unit (Hiperbaric 300, Hiperbaric S.A., Burgos, Spain) according to the procedure described by Opazo-Navarrete et al. (28). Samples were treated at 300, 400, 500, and 600 MPa for 3, 6, and 9 min, and subsequently compared with untreated samples. Water served as the pressure-transmitting medium, with a pressurization rate of 3 MPa/s; while decompression was completed in less than 3 s.

After HHP treatment, samples were immediately frozen at −80 °C and freeze-dried for 72 h using a freeze-dryer (FreeZone 6 Plus, Labconco, Kansas City, Missouri, USA). The dried material was ground with a Fritsch Mill Pulverisette 14 (Idar-Oberstein, Germany) at 6,000 rpm, then passed through a 200 μm sieve, and stored at −20 ± 1 °C until further analysis.

### 2.6 Fourier-transform infrared spectroscopy with attenuated total reflectance (FTIR-ATR)

The infrared spectra were recorded using an FTIR spectrometer (IRSpirit, Shimadzu Corporation Pte. Ltd., Kyoto, Japan), following a protocol adapted from Cepero-Betancourt et al. (29). Measurements were conducted in absorbance mode by collecting 128 scans at a resolution of 4 cm^−1^ over the spectral range of 4,000–400 cm^−1^. For measurement, approximately 10 mg of each sample was analyzed at ambient temperature utilizing an attenuated total reflectance (ATR) accessory. The samples were directly placed onto the ATR crystal and gently pressed with a flat-tip device to ensure proper contact prior to data acquisition.

For the assessment of protein secondary structure, the amide I region (1,700–1,600 cm^−1^) was designated. The spectral data were pre-processed by employing a Savitzky-Golay algorithm (13–point), followed by the normalized second-derivative spectra utilizing LabSolutions IR software (Shimadzu, Kyoto, Japan).

To deconvolute overlapping bands within the amide I region, Gaussian curve fitting was applied to the second-derivative spectra using Origin software (version 2019b, OriginLab Corporation, Northampton, USA). The relative proportions of secondary structure elements were estimated by calculating the area associated with each assigned peak. All measurements were performed in triplicate to ensure analytical reproducibility.

### 2.7 Simulated *in vitro* gastrointestinal digestion

The *in vitro* gastrointestinal digestion was conducted in accordance with the standardized INFOGEST protocol, as outlined by Opazo-Navarrete et al. (28). Prior to the digestion process, the activity levels of digestive enzymes and bile salt concentrations were assessed in accordance with the procedures specified in the harmonized protocol (30).

For each assay, 0.04 g of protein sample was dispersed in distilled water. The gastric phase was initiated by adding simulated gastric fluid (pH 3.0, 37 °C) containing pepsin at a final activity of 2,000 U/ml of digesta, followed by incubation for 120 min under continuous agitation at 100 rpm. Subsequently, the intestinal phase was initiated by adding simulated intestinal fluid (pH 7.0), supplemented with pancreatin (100 U trypsin activity/ml of digesta) and bile salts at a final concentration of 10 mmol/L. Samples were then incubated for 180 min at 37 °C under constant agitation at 300 rpm.

The gastric phase was concluded by elevating the pH to 7.0 through the addition of 1 mol/L NaOH. Subsequently, intestinal digestion was stopped by introducing the protease inhibitor 4-(2-aminoethyl) benzenesulfonyl fluoride (AEBSF; Pefabloc^®^, 500 mmol/L; Roche, Basel, Switzerland).

### 2.8 Degree of hydrolysis (DH)

The DH was quantified using the o-phthaldialdehyde (OPA) assay following the method described by Opazo-Navarrete et al. (31). The OPA reagent (100 ml) was freshly prepared on the day of analysis and stored in a light-protected container. A standard calibration curve was made using L-serine at concentrations ranging from 50 to 200 mg/L. For each measurement, 200 μl of sample or standard solution was combined with 1.5 ml of OPA reagent. After incubation for 3 min at ambient temperature, absorbance was recorded at 340 nm using an HT Multi-Detection Microplate Reader (Biotek Instruments Inc., Winooski, VT, USA).

DH values for both digested and undigested samples were calculated by converting absorbance readings to free amino group concentrations (mmol/L) using the calibration curve. The initial free amino group content of the undigested samples was subtracted to correct for baseline levels. The results were expressed as serine amino equivalents (N-terminal serine) to represent the concentration of free amino groups. DH values were then calculated using the Equations 1 and 2:


$$\begin{array}{l} DH\left(\%\right)=\frac{h}{h_{tot}}\times 100 \end{array}$$ (1)

$$\begin{array}{l} h\;=\;\frac{Serine\;{NH}_{2}-\beta }{\alpha } \end{array}$$ (2)

For the estimation of DH values, the constants α and β were fixed at 1 and 0.4, respectively, as previously reported by Opazo-Navarrete et al. (32). The total peptide bond content (*h*_*tot*_) was calculated from the amino acid profile of the fava bean protein, resulting in a value of 6.94 meq/g protein. All experimental measurements were conducted in triplicate.

### 2.9 Protein solubility

The protein solubility (%) of both HHP-treated and untreated FBPI samples was assessed following the protocol outlined by Opazo-Navarrete et al. (31). Dispersions were prepared at a concentration of 3% (w/v) in purified water, and the pH was adjusted to 7.0 using 0.1 M HCl or 0.1 M NaOH as necessary. The dispersions were maintained under continuous stirring at ambient temperature for 1 h.

Subsequently, sample concentrations were subjected to centrifugation at 15,520 × *g* for 15 min at 20 °C (GYROZEN 1580R, Daejeon, Korea) to separate soluble and insoluble fractions. The supernatant was carefully collected, stored at −20 °C, and then freeze-dried (Liobras, Liotop LP1280, São Carlos, Brazil). The resulting dried fraction was weighed, and its protein content was quantified utilizing the Dumas method (Dumatherm^®^ N Pro, Königswinter, Germany).

Protein solubility (%) was estimated according to Equation 3 and expressed as the percentage of soluble protein relative to the total protein content of the initial sample.


$$\begin{array}{l} Protein\;solubility\;\left(\%\right)=\frac{mass\;supernatant\;\left(mg\right)\times protein\;content\;supernatant\;\left(\%\right)}{sample\;mass\;\left(mg\right)\times protein\;content\;\left(\%\right)\;}\times 100 \end{array}$$ (3)

All experimental measurements were conducted in triplicate.

### 2.10 Emulsifying capacity (EC) and emulsion stability (ES)

The emulsifying properties of FBPI samples, including EC and ES, were assessed in accordance with a methodology adapted from Opazo-Navarrete et al. (29). Specifically, 0.2 g of FBPI sample was dispersed in 20 ml of purified water to prepare a 1% (w/v) suspension within a 50 ml centrifuge tube. The mixture was agitated for 1 h at ambient temperature utilizing an orbital shaker (Multi Reax, Heidolph Instruments, Schwabach, Germany), and subsequently stored at 4 °C overnight.

Before emulsification, the pH was adjusted to 7.0, and an equal volume of sunflower oil was introduced to attain a 1:1 (v/v) aqueous-to-oil ratio. The sample was homogenized at 10,000 rpm for 2.5 min using an Ultra-Turrax homogenizer (IKA Werke GmbH & Co. KG, Staufen, Germany). The resultant emulsion was allowed to stand at ambient temperature for 1 h and was subsequently transferred to graduated cylinders.

The total height of the emulsion (H_T_) and the height of the emulsified layer (H_EL_) were measured at 0 and 24 h. Emulsifying capacity after 24 h (EC_24_), estimated according to Equation 4, was expressed as the percentage ratio of H_EL_ to H_T_, while, calculated according to Equation 5, ES was calculated as the ratio between EC_24_ and the initial emulsifying capacity (EC_0_).


$$\begin{array}{l} EC\left(\%\right)=\frac{H_{EL}}{H_{T}}\times 100 \end{array}$$ (4)

$$\begin{array}{l} ES\left(\%\right)=\frac{{EC}_{24}}{{EC}_{0}}\times 100 \end{array}$$ (5)

All experimental measurements were conducted in triplicate.

### 2.11 Foaming properties

Foaming properties, including foaming capacity (FC) and foaming stability (FS), were evaluated using a protocol adapted from Domínguez-Valencia et al. (33) with minor modifications. Protein dispersions were prepared by dissolving 1 g of FBPI in 100 ml of purified water, after which the pH was adjusted to 7.0. The suspension was stirred for 1 h using a stirrer to ensure complete hydration.

Following hydration, the dispersions (initial volume, V1 = 100 ml) were transferred into a 250 ml graduated beaker and homogenized at 17,500 rpm for 2 min using an Ultra-Turrax disperser. The volume of foam produced immediately post-homogenization (V0) was documented to assess foaming capacity (FC), whereas the foam volume remaining after a 30 min of rest (V30) was measured to evaluate foaming stability (FS).

Foaming capacity and stability were determined according to Equations 6 and 7, by assessing the change in volume prior to and following homogenization, utilizing the relevant equations.


$$\begin{array}{l} FC\;\left(\%\right)=\frac{V_{0}}{V_{1}}\times 100 \end{array}$$ (6)

$$\begin{array}{l} FS\;\left(\%\right)=\frac{V_{30}}{V_{0}}\times 100 \end{array}$$ (7)

All experimental measurements were conducted in triplicate.

### 2.12 Water holding capacity (WHC)

Water holding capacity (WHC) was assessed utilizing a modified protocol derived from Melchior et al. (34). 1.0 g of FBPI samples were transferred into a Vivaspin 20 centrifugal filter unit (GE Healthcare Bio-Sciences AB, Uppsala, Sweden) and subjected to centrifugation at 4,000 x *g* for 20 min. The mass of the samples was documented prior to or subsequent to centrifugation. The WHC values were determined based on the differential mass, according to Equation 8:


$$\begin{array}{l} WHC\;\left(\%\right)=\frac{M_{1}-M_{2}}{M_{1}}\times 100 \end{array}$$ (8)

where M_1_ denotes the initial mass of total water in the sample prior to centrifugation (g), whereas M_2_ refers to the water expelled from the sample as a consequence of centrifugation (g). All experimental measurements were conducted in triplicate.

### 2.13 Statistical analysis

The results are presented as mean values ± standard deviation, derived from three independent replicates for each analysis. Statistical assessment was performed utilizing the Statgraphics Centurion XVI software (Statistical Graphics Corporation, Herndon, VA, USA). Variations among treatments were evaluated through one-way analysis of variance (ANOVA), followed by Duncan's multiple range test to identify significant differences between means at a confidence level of *p* < 0.05.

Furthermore, a Pearson correlation analysis was conducted to investigate the relationships between the relative proportions of protein secondary structure components and the DH obtained post-*in vitro* digestion. Correlation coefficients (r) and their respective *p*-values were computed to evaluate the strength and statistical significance of these correlations.

All experiments were conducted utilizing three independent sample preparations, with each analytical determination performed in triplicate.

## 3 Results and discussion

### 3.1 Chemical characteristics of FBPI

The chemical composition of the FBPI, presented in Table 1, exhibits a high protein purity (89.6 ± 0.52%) and efficient fractionation, aligning with previously reported values for legume protein isolates (85–94%) (10, 27, 35). The absence of lipids and dietary fiber indicates the effective removal of non-protein components during processing, which is advantageous for both digestibility and product stability (4, 36). The ash content remains within the typical range for legume isolates, while the residual carbohydrates, determined by difference, likely correspond to minor quantities of starch or oligosaccharides. Overall, this compositional profile substantiates the suitability of FBPI as a highly purified protein ingredient for subsequent processing.

**Table 1:** Chemical composition for fava bean protein isolate (FBPI).

Component	Content (%)
Protein	89.6 ± 0.52
Lipids	0
Dietary fiber	0
Ash	3.49 ± 0.11
Carbohydrates (NSE)^*^	6.96 ± 0.15

^*^Carbohydrates were determined by difference.

The amino acid profile of the FBPI is presented in Table 2, showing a well-balanced composition and notably superior profile than many other cereals and pulse sources (1, 3) with a predominance of essential amino acids (EAAs), particularly leucine (9.53 ± 0.55%), lysine (5.87 ± 0.49%), and valine (4.72 ± 0.37%). The high lysine content is especially relevant, as it complements cereal-based proteins that are typically deficient in this amino acid, reinforcing the nutritional value of FBPI for plant-based formulations (5). However, sulfur-containing amino acids (SAA, Met + Cys) were present in low amounts (0.73 ± 0.06%), which is consistent with the known limitation of legume proteins and may represent a nutritional constraint if not complemented with other protein sources (35). This deficiency is primarily ascribed to the fact that the principal storage proteins, namely globulins (legumin and vicilin), inherently possess a lower concentration of these residues in comparison to the albumin fraction (14).

**Table 2:** Amino acid profile of fava bean protein isolate (FBPI).

Amino acid (AA)	Content (%)
*Essential amino acids*	
His	2.27 ± 0.21
Ile	4.41 ± 0.25
Leu	9.53 ± 0.55
Lys	5.87 ± 0.49
SAA (Met + Cys)	0.73 ± 0.06
Met	0.13 ± 0.01
AAA (Phe + Tyr)	7.59 ± 0.69
Phe	4.62 ± 0.33
Thr	2.93 ± 0.21
Trp	0.38 ± 0.03
Val	4.72 ± 0.37
*Conditionally essential amino acids*	
Arg	8.7 ± 0.63
Cys–Cys	0.60 ± 0.04
Glyc	3.34 ± 0.34
Pro	4.15 ± 0.16
Tyr	2.96 ± 0.16
*Non–essential amino acids*	
Ala	3.93 ± 0.24
Asp	8.23 ± 0.62
Glu	14.7 ± 1.16
Ser	4.34 ± 0.15
Hyp	< 0.01
Tau	0.25 ± 0.02

Among the conditionally essential amino acids, arginine was identified at elevated levels (8.7 ± 0.63%), characteristic of legume proteins, and has been linked to potential health benefits, including cardiovascular support (37). Proline (4.15 ± 0.16%) and glycine (3.34 ± 0.34%) were also detected in significant quantities, potentially contributing to the structural and functional properties of the protein matrix.

Concerning non-essential amino acids, glutamic acid (14.7 ± 1.16%) and aspartic acid (8.23 ± 0.62%) were identified as the most prevalent components, which is characteristic of plant proteins. These amino acids may influence both flavor, specifically umami-related attributes, and techno-functional properties such as solubility and water-binding capacity (10, 38). The minimal levels of hydroxyproline (< 0.01%) corroborate the absence of collagen-type proteins, as anticipated for plant-derived isolates.

Overall, the compositional and amino acid profiles indicate that FBPI is a high-protein source, with minimal presence of non-protein components, and an amino acid composition superior to other cereals and pulse sources.

### 3.2 Protein structure

Protein structural characterization is essential to understand the effects of processing treatments on protein functionality and nutritional quality. In this study, structural modifications induced by the HHP treatment were evaluated at different levels of organization. FTIR was used to assess changes in secondary structure, while the determination of free sulfhydryl (SH) groups provided insight into alterations in tertiary structure and disulfide bond dynamics. Together, these analyses allow a comprehensive evaluation of protein conformational changes associated with processing.

#### 3.2.1 FTIR-ATR analysis

**Figure 1:**
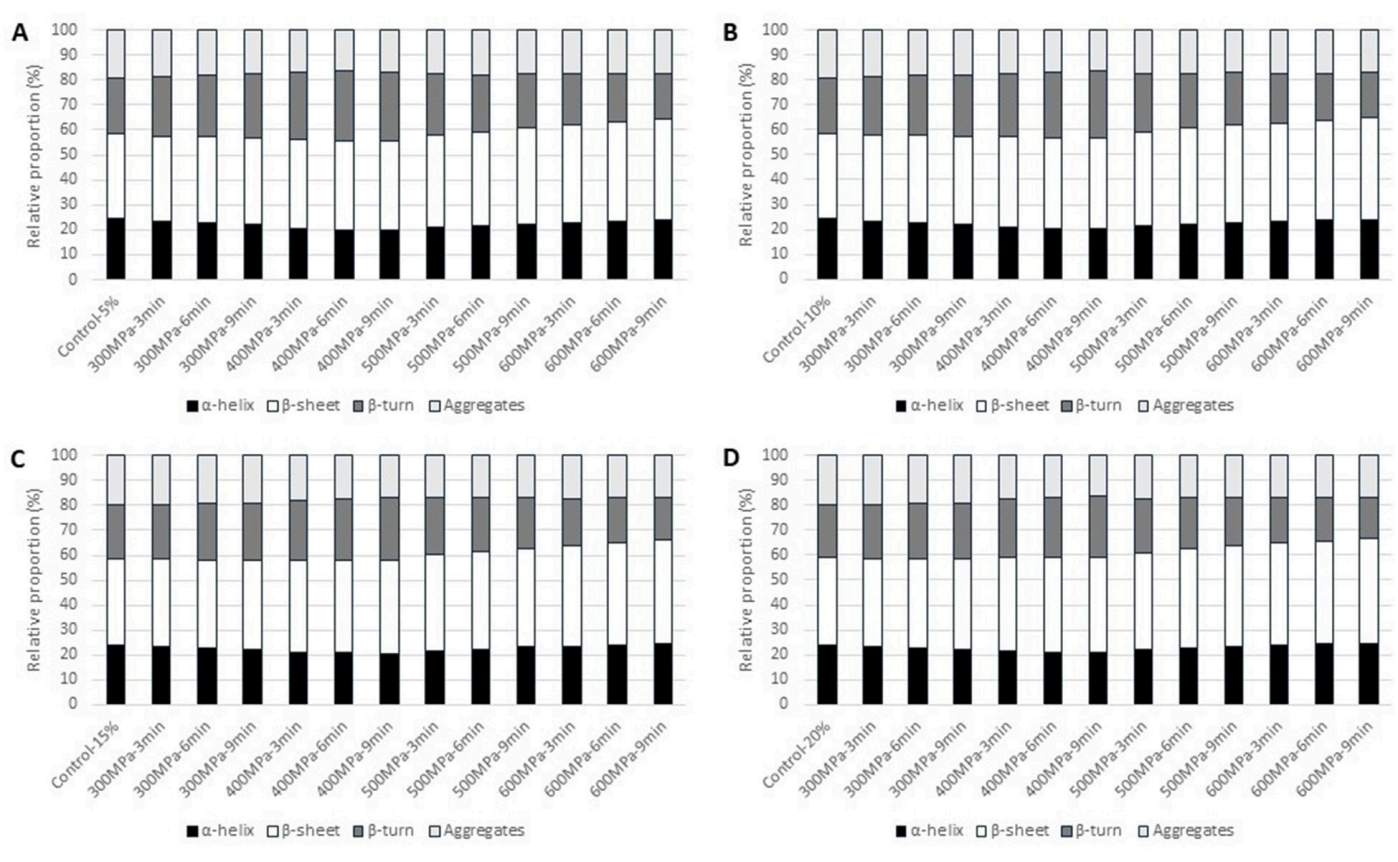
Effect of high hydrostatic pressure (300–600 MPa, 3–9 min) on the secondary structure composition of FBPI dissolutions at different concentrations: **(A)** 5%, **(B)** 10%, **(C)** 15%, and **(D)** 20%, as determined by FTIR-ATR analysis.

Figure 1 shows the effect of HHP treatments (300–600 MPa, 3–9 min) on the secondary structure composition of FBPI at different protein concentrations (5–20%), as determined by FTIR-ATR. In general, the untreated samples exhibited a predominance of β-sheet structures, followed by α-helix and β-turn, which is characteristic of legume globulin-type proteins.

The modifications observed in the secondary structure of FBPI under HHP (Figure 1) are consistent with thermodynamic principles, in which pressure favors conformations with lower partial molar volume through the disruption of non-covalent interactions such as hydrophobic and electrostatic forces (39). These structural changes are in line with those reported for other legume proteins, which share a globulin-based structure (legumin and vicilin), where HHP induces partial unfolding followed by reorganization into intermolecular β-sheet structures associated with aggregation (40, 41).

At a protein concentration of 5% (Figure 1A), HHP induced notable structural modifications, characterized by a gradual decline in α-helix content from 24.8% in the control to a minimum of 19.8% at 400 MPa (6 min), followed by a partial recovery at higher pressures (23.9% at 600 MPa, 9 min). Concurrently, β-sheet structures increased from 33.5% to 40.5%, indicating protein unfolding and subsequent structural reorganization. Furthermore, β-turn structures initially increased (up to 28.1% at 400 MPa), suggesting the formation of intermediate conformations, and later decreased at higher pressures, likely due to their conversion into more stable β-sheet configurations. Structures related to aggregation exhibited only minor variation (~16–19%), implying that aggregation may occur in more ordered conformations rather than as large, disordered aggregates. These observations are consistent with previous research indicating that pressures exceeding 400 MPa can induce irreversible conformational transitions in legume proteins.

At a concentration of 10% (Figure 1B), similar trends were observed; however, the magnitude of these changes was reduced. The α-helix fraction decreased from 24.5% to approximately 20.1% at 400 MPa, followed by recovery at higher pressures, while β-sheet structures increased from 34.0% to approximately 41.0%. Aggregate formation remained relatively stable (~17–19%), thereby confirming that increasing protein concentration restricts molecular mobility, consequently reducing susceptibility to pressure-induced unfolding and limiting the formation of new intermolecular interactions (42, 43).

At elevated concentrations (15% and 20%, Figure 1C, D), FBPI demonstrated enhanced structural stability under HHP conditions. Although some degree of structural rearrangement was still observed, such as an increase in β-sheet content from 34.5% to 41.6% (15%) and from 35.0% to 42.0% (20%), the changes in α-helix content were less pronounced, and the overall distribution of secondary structures remained relatively stable. Additionally, β-turn structures showed a gradual decrease (e.g., from 21.5% to 17.2% at 15%), suggesting their role as intermediate conformations during structural transitions. Aggregate-related structures remained nearly constant (~16.7–20.0%), indicating limited formation of new aggregates. This increased resistance can be ascribed to high molecular density effects and reduced solvent accessibility, which restrict conformational flexibility and impede unfolding processes (44). In these concentrated systems, proteins are densely packed, forming a compact matrix that restricts structural transitions even when subjected to elevated pressures.

Furthermore, the limited aggregation observed at higher concentrations indicates that the formation of pressure-induced intermediates is constrained, thereby inhibiting the development of extensive aggregate networks (45). This observation aligns with findings in other legume proteins, where elevated protein concentrations necessitate more severe processing conditions to elicit comparable structural modifications (14).

Consequently, the findings illustrate that HHP induces concentration-dependent structural alterations in FBPI. While reduced concentrations facilitate partial unfolding and aggregation, elevated protein concentrations enhance structural rigidity and stability. This highlights the pivotal role of matrix organization in dictating protein responsiveness to HHP and suggests that FBPI-based systems with high solids content may preserve their structural integrity during pressure processing.

#### 3.2.2 SH groups

**Figure 2:**
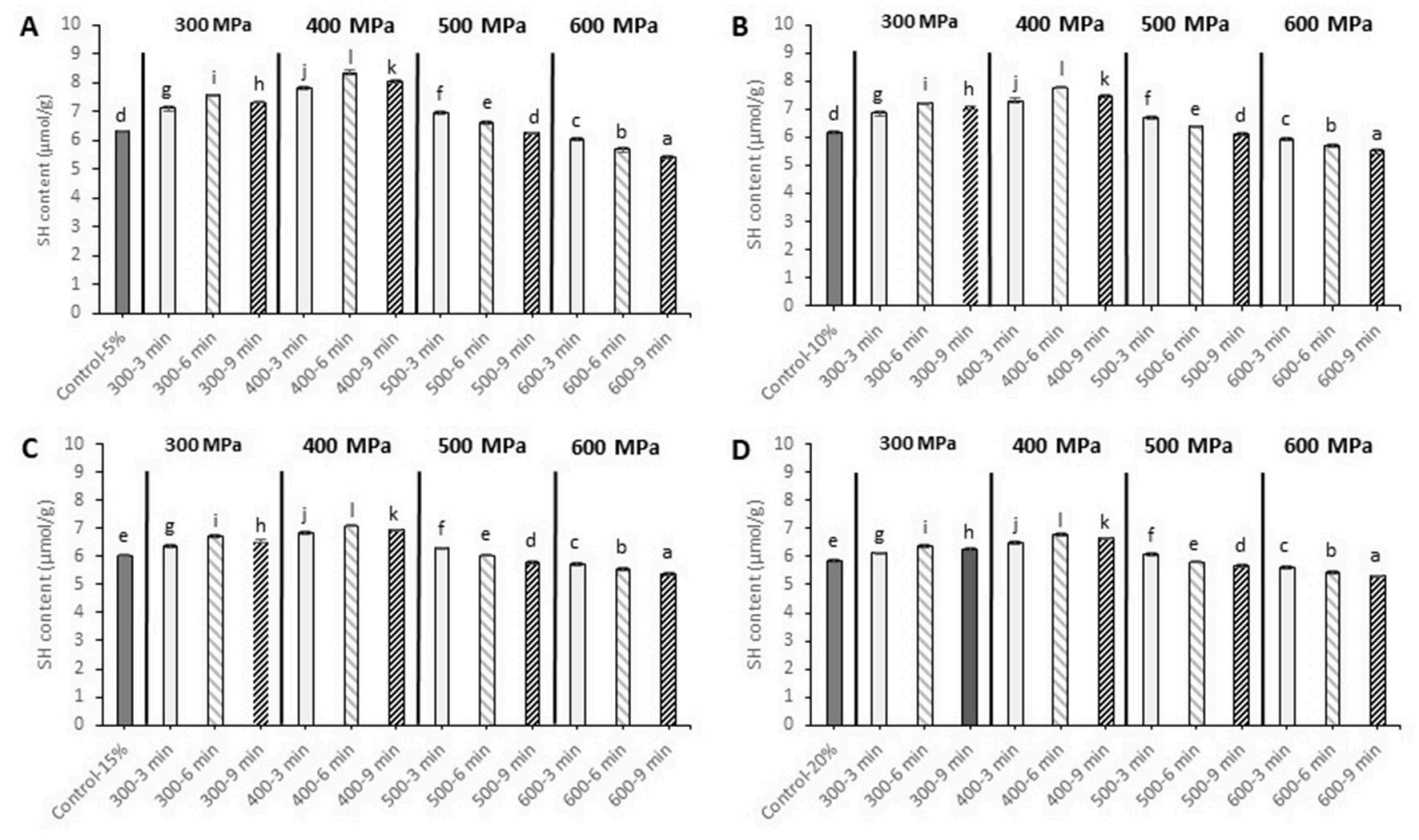
Free sulfhydryl groups (SH) of fava bean protein isolate (FBPI), for untreated (control) and HP-treated (300–600 MPa) samples at different processing times (3–9 min) and protein concentrations. **(A)** 5%, **(B)** 10%, **(C)** 15%, and **(D)** 20%. Results are means and standard deviations of replicates. Different letters at the top of a column indicate significant differences (*p* < 0.05) between samples (with the same protein concentration) treated at different pressure levels and times.

The effect of HHP on the SH groups of FBPI at different protein concentrations is presented in Figure 2. Overall, the SH content demonstrated a pressure-dependent pattern, with fluctuations significantly affected by both protein concentration and the degree of treatment.

The initial increase in SH content observed at 5% and 10% protein concentrations between 300 and 400 MPa aligns with the pressure-induced unfolding of globular proteins. This unfolding process results in the expansion of tertiary structures, thereby exposing previously concealed SH groups located within the hydrophobic core (46). At a concentration of 5%, SH values increased from 6.30 μmol/g in the control to a maximum of 8.35 μmol/g at 400 MPa for 6 min, while at a concentration of 10%, values rose from 6.15 to 7.78 μmol/g under identical conditions.

In fava beans, this phenomenon is notably linked to the 11S legumin fraction, which encompasses the cysteine-free 7S vicilin fraction (10). The exposure of these reactive groups signifies a disruption of the tertiary conformation, while partial secondary structures are preserved, indicative of an intermediate partially unfolded state, a conclusion further corroborated by FTIR analysis (47).

As pressure exceeds 400 MPa and holding time is prolonged, a consistent decline in SH content is noted, suggesting that these reactive groups partake in SH/S–S exchange reactions or the formation of novel intermolecular disulfide bonds. At a concentration of 5%, SH values decreased from 8.35 μmol/g (400 MPa, 6 min) to 5.42 μmol/g at 600 MPa for 9 min, with comparable trends observed at 10% (from 7.78 to 5.50 μmol/g). This transition signifies a shift from unfolding to aggregation, corroborated by the increase in β-sheet structures and aggregate-related formations. Under these conditions, exposed SH groups function as reactive intermediates that facilitate the development of stable, high-molecular-weight networks, indicating irreversible structural reorganization (45, 46).

In addition to pressure, treatment time exerted a secondary yet noticeable effect. At intermediate pressures (300–400 MPa), extending the holding time from 3 to 6 min generally augmented SH groups exposure (e.g., from 7.10 to 7.55 μmol/g at 300 MPa for 5%), although slight reductions were observed at 9 min, indicating the onset of reaggregation. At higher pressures (≥500 MPa), prolonged treatment times further contributed to the decrease in SH content, signifying increased intermolecular bonding. Nevertheless, these variations were relatively insignificant compared to the influence of pressure, thereby confirming that HHP-induced structural modifications are predominantly driven by pressure.

In contrast, the reduced stability in SH content at 15% and 20% protein concentrations highlights a clear concentration-dependent baroresistance. For instance, at 15%, SH content increased only from 6.05 to 7.10 μmol/g at 400 MPa, while at 20%, the maximum value reached was 6.80 μmol/g. Moreover, the subsequent decrease at high pressures was less pronounced, with final values remaining above 5.30 μmol/g even at 600 MPa. This limited variation indicates restricted protein unfolding under crowded conditions. In these systems, molecular crowding and restricted molecular mobility act as mechanical barriers to the volume expansion required for extensive protein unfolding, thereby reducing the exposure of SH groups. As a result, the formation of pressure-induced intermediate states is constrained, and reactive groups remain largely inaccessible. This behavior is consistent with the FTIR results, where secondary structure elements remained largely unchanged, confirming that high-concentration FBPI forms a compact and structurally robust matrix under HHP.

### 3.3 Protein digestibility

The DH of FBPI was significantly influenced by pressure, holding time, and protein concentration (Table 3). Overall, DH exhibited a biphasic response to HHP treatment, increasing at moderate pressures (300–400 MPa) and decreasing at higher pressures (500–600 MPa). The magnitude of these changes was more pronounced at lower protein concentrations, whereas concentrated systems (15–20%) showed a reduced response to pressure treatment.

**Table 3:** Degree of hydrolysis (DH) of protein dispersions.

Pressure (MPa)	Time (min)	5%	10%	15%	20%
0.1	–	69.5 ± 0.29^aD^	67.7 ± 0.55^aC^	65.7 ± 0.30^aB^	63.2 ± 0.26^aA^
300	3	75.2 ± 0.25^dD^	72.4 ± 0.50^cC^	69.2 ± 0.31^bB^	66.4 ± 0.31^bA^
	6	76.7 ± 0.21^dD^	73.4 ± 0.36^dC^	70.2 ± 0.35^cB^	67.3 ± 0.23^cA^
	9	78.2 ± 0.26^fD^	74.1 ± 0.57^fC^	71.3 ± 0.35^dB^	68.7 ± 0.26^dA^
400	3	86.3 ± 0.29^lD^	80.7 ± 0.40^kC^	75.6 ± 0.20^gB^	71.8 ± 0.15^gA^
	6	85.7 ± 0.17^kD^	80.5 ± 0.17^jkC^	76.2 ± 0.12^hB^	72.6 ± 0.31^hA^
	9	84.9 ± 0.26^jD^	79.9 ± 0.21^jC^	76.8 ± 0.25^iB^	73.2 ± 0.32^iA^
500	3	82.1 ± 0.20^iD^	77.8 ± 0.29^iC^	74.5 ± 0.17^fB^	71.6 ± 0.25^gA^
	6	80.6 ± 0.06^hD^	76.5 ± 0.40^hC^	72.9 ± 0.26^eB^	70.3 ± 0.15^fA^
	9	79.1 ± 0.20^gD^	75.1 ± 0.35^gC^	71.6 ± 0.36^dB^	69.5 ± 0.35^eA^
600	3	76.4 ± 0.21^eD^	73.7 ± 0.26^efC^	71.2 ± 0.31^dB^	69.5 ± 0.31^eA^
	6	74.6 ± 0.21^cD^	72.9 ± 0.30^cdC^	70.5 ± 0.20^cB^	68.9 ± 0.35^dA^
	9	72.8 ± 0.36^bD^	71.2 ± 0.25^bC^	69.1 ± 0.30^bB^	67.6 ± 0.26^cA^

Means with different capital letters in the same row indicate significant differences among protein concentrations (*p* < 0.05). Means with different lowercase letters in the same column indicate significant differences among treatments (*p* < 0.05).

The observed increase in DH with pressure up to 400 MPa is consistent with the structural modifications detected by FTIR analysis and SH group measurements, which indicated partial protein unfolding and increased exposure of reactive sites. From a thermodynamic perspective, HHP shifts the equilibrium toward a molten globule-like state, characterized by preserved secondary structure but disrupted tertiary organization, resulting in increased conformational flexibility (17, 45). This structural transition facilitates the access of digestive enzymes such as pepsin and trypsin, thereby improving hydrolysis efficiency. Similar trends have been reported in other pulse proteins, where moderate pressures (200–400 MPa) enhance *in vitro* digestibility by loosening the compact globulin structure (3, 36).

Nonetheless, the observed progressive decline in DH beyond 400 MPa signifies that excessive pressure facilitates irreversible protein aggregation. In accordance with Le Chatelier's principle, pressure encourages conformations with reduced volume, resulting in the collapse of internal cavities and the exposure of hydrophobic and SH groups (14). At elevated pressures (500–600 MPa), these reactive species contribute to the formation of large aggregates stabilized by intermolecular β-sheet structures and disulfide cross-links (26, 48). Such aggregates impede enzyme accessibility by shielding cleavage sites and restricting diffusion, thereby accounting for the observed decrease in DH.

This interpretation aligns with the FTIR and SH results, which demonstrated increased aggregation markers under high-pressure conditions. Holding time exerted a secondary yet significant effect. At 300 MPa, extending the treatment time from 3 to 9 min promoted gradual unfolding and improved DH, indicating that moderate pressures necessitate longer exposure durations to attain maximal structural disruption (17).

In contrast, at pressures ≥400 MPa, prolonged treatment resulted in a sligh but consistent decrease in DH, indicating that extended exposure favors the reorganization of unfolded proteins into more stable, enzyme-resistant aggregates. This suggests the existence of a narrow processing window in which unfolding is maximized while aggregation is minimized. Protein concentration played a critical role in modulating these effects, as DH consistently decreased with increasing concentration (5%−20%). This behavior can be explained by the increased probability of protein-protein interactions at higher concentrations, which promotes the formation of compact structures that restrict enzymatic access (14, 42). In concentrated systems, the higher frequency of intermolecular interactions promotes the formation of dense aggregates or gel-like structures during HHP treatment (43, 49). These compact matrices hinder enzyme diffusion and restrict access to proteolytic sites, resulting in lower hydrolysis efficiency. Accordingly, the pressure resistance observed at 15% and 20%, supported by FTIR and SH analyses, directly correlates with reduced digestibility.

Therefore, moderate pressures (around 400 MPa) combined with short to intermediate holding times optimize the digestibility of FBPI by balancing protein unfolding and aggregation phenomena. Conversely, higher pressures, extended treatment times, and increased protein concentration promote structural stabilization and aggregation, thereby reducing enzymatic hydrolysis. These findings underscore the significance of tailoring HHP conditions in accordance with matrix composition to enhance the nutritional quality of plant-based proteins.

Furthermore, Pearson correlation analysis demonstrated significant associations between modifications in protein secondary structure and *in vitro* digestibility (DH), thereby elucidating the structural determinants that influence enzymatic susceptibility. Notably, DH exhibited a strong positive correlation with the relative content of α-helix and β-turn structures, while a negative correlation was observed with β-sheet structures. These findings suggest that increased flexibility and reduced order in protein conformations enhance enzymatic accessibility through facilitated exposure of peptide bonds. Conversely, the formation of β-sheet-rich structures, which are typically associated with protein aggregation, impairs proteolytic efficiency. This behavior aligns with the structural transitions induced by HHP, wherein moderate pressures promote partial unfolding and increased conformational mobility, thereby augmenting digestibility. Conversely, at elevated pressures, the stabilization of intermolecular β-sheet structures and aggregate formation reduces enzyme accessibility, resulting in lower DH values. In summary, these results confirm that protein digestibility is predominantly governed by the equilibrium between structural flexibility and aggregation, establishing a direct relationship between structure and digestibility in FBPI systems subjected to HHP.

### 3.4 Functional properties

#### 3.4.1 Protein solubility

**Figure 3:**
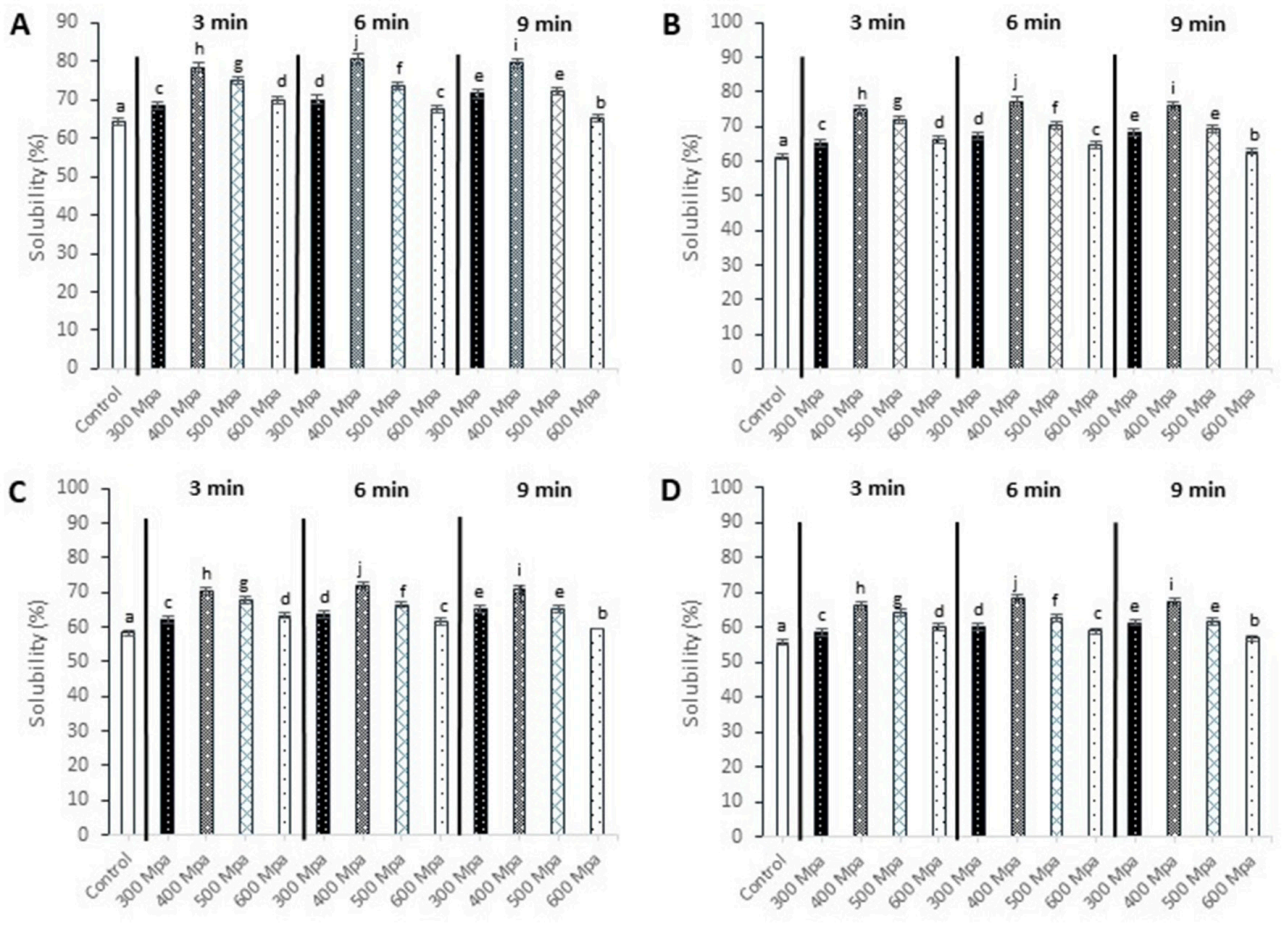
Effect of high hydrostatic pressure (HHP) and holding time on the solubility of fava bean protein isolate (FBPI) at different protein concentrations. **(A)** 5%, **(B)** 10%, **(C)** 15%, and **(D)** 20%.

Protein solubility at pH 7.0 is presented in Figure 3. Solubility values were significantly affected by pressure level, holding time, and protein concentration (*p* < 0.05). Overall, protein solubility exhibited a biphasic response to HHP treatment, increasing with pressure up to 400 MPa, followed by a decrease at higher pressures (500–600 MPa), indicating a balance between protein unfolding and aggregation phenomena.

At lower protein concentrations (5% and 10%), the notable increase in solubility observed at pressures up to 400 MPa can be attributed to the partial dissociation of multimeric storage proteins, particularly the 11S legumin and 7S vicilin fractions (20, 27). This phenomenon aligns with the structural modifications identified through FTIR analysis and SH group measurements, which indicate partial protein unfolding under moderate pressure conditions. Consequently, protein–solvent interactions are enhanced, leading to an increased proportion of soluble protein. This phenomenon suggests a transition toward more flexible conformations without complete structural disintegration, a behavior that is frequently reported in pulse proteins subjected to intermediate levels (16, 45).

In contrast to digestibility trends, where enzymatic accessibility is the primary factor, solubility seems to be more significantly influenced by the equilibrium between protein-protein and protein-water interactions. Within this framework, the enhanced solubility observed at moderate pressures indicates that the disruption of intermolecular associations outweighs aggregation phenomena, at least within this pressure spectrum. This differentiation underscores that structural alterations caused by HHP do not invariably result in proportional changes across functional properties.

When the pressure exceeds 400 MPa, the decline in solubility indicates a transition toward aggregation-dominated behavior. At these intensities, the exposure of hydrophobic regions and reactive groups facilitates stronger intermolecular associations, resulting in the formation of insoluble aggregates. These structures are stabilized through a combination of hydrophobic interactions, hydrogen bonding, and disulfide cross-linking, thereby reducing the capacity of proteins to remain dispersed in aqueous systems (17, 50). FTIR results corroborate this interpretation, demonstrating an increase in β-sheet and aggregate-related structures under high-pressure conditions, which are typically linked to reduced solubility.

The impact of holding time further corroborates this structural interpretation. At 300 MPa, the gradual augmentation of solubility over time indicates that protein unfolding advances gradually, necessitating adequate residence time to fully expose interaction sites with water. Nonetheless, at elevated pressures (≥400 MPa), prolonged treatment encourages the reorganization of unfolded chains into more ordered and less soluble assemblies. This suggests that time functions as a kinetic parameter, initially supporting structural disruption but later permitting aggregation pathways to predominate as exposure persists.

Protein concentration adds a further dimension of complexity, as solubility consistently reduces with escalating solids content. This behavior is not merely indicative of limited solvent availability but is also associated with an increased propensity for intermolecular interactions within concentrated systems, thereby favoring the formation of interconnected protein networks. Such networks reduce the effective surface area available for hydration and facilitate phase separation phenomena.

At 15% and 20%, the minor variations in solubility across different pressure treatments indicate that these systems demonstrate increased structural stability when subjected to pressure. In such dense matrices, conformational rearrangements are restricted, thereby limiting both the degree of unfolding and subsequent aggregation. This constrained structural response aligns with FTIR and SH observations, where higher concentrations displayed reduced sensitivity to pressure-induced alterations. Accordingly, the functionality of FBPI under HHP appears to be highly dependent on the matrix, with dilute systems being more susceptible to pressure-induced modifications, whereas concentrated systems maintain a more stable and less soluble structure.

#### 3.4.2 Emulsifying properties of HHP-treated FBPI samples

The EC and ES of HHP-treated FBPI samples are presented in Table 4. For the untreated control samples, the progressive decline indicates that increasing protein concentration negatively affects interfacial performance, likely due to reduced molecular mobility and intensified protein-protein interactions in concentrated systems.

**Table 4:** Emulsifying properties of fava bean protein isolate (FBPI) samples subjected to high hydrostatic pressure (HHP).

Parameter	Pressure (MPa)	Time (min)	5%	10%	15%	20%
EC (%)	0.1	–	74.7 ± 0.67^dD^	72.1 ± 0.49^dC^	67.8 ± 0.64^cB^	63.9 ± 0.66^cA^
	300	3	77.8 ± 0.40^eD^	74.4 ± 0.38^eC^	70.5 ± 0.51^dB^	66.8 ± 0.79^deA^
		6	79.2 ± 0.55^fD^	76.2 ± 0.46^fC^	71.2 ± 0.78^deB^	67.9 ± 0.57^efA^
		9	80.1 ± 0.71^fgD^	77.5 ± 0.42^fgC^	71.8 ± 0.64^eB^	68.5 ± 0.67^fgA^
	400	3	83.1 ± 0.45^hD^	80.2 ± 0.45^hC^	75.9 ± 0.75^gB^	72.1 ± 0.87^hA^
		6	85.3 ± 0.46^iD^	82.2 ± 0.58^iC^	77.6 ± 0.53^hB^	73.8 ± 0.81^iA^
		9	86.4 ± 0.62^iD^	83.1 ± 0.49^iC^	78.4 ± 0.50^hB^	74.7 ± 0.72^iA^
	500	3	80.4 ± 0.36^gD^	77.8 ± 0.65^gC^	73.2 ± 0.55^fB^	69.4 ± 0.68^gA^
		6	77.4 ± 0.68^eD^	74.4 ± 0.68^eC^	70.2 ± 0.75^dB^	66.2 ± 0.68^dA^
		9	74.9 ± 0.61^dD^	71.5 ± 0.59^dC^	67.1 ± 0.85^cB^	63.8 ± 0.85^cA^
	600	3	72.9 ± 0.61^cD^	69.7 ± 0.68^cC^	66.9 ± 0.59^cB^	64.1 ± 0.83^cA^
		6	69.2 ± 0.47^bD^	65.4 ± 0.79^bC^	62.6 ± 0.78^bB^	59.6 ± 0.70^bA^
		9	67.2 ± 0.38^aD^	63.9 ± 0.51^aC^	59.8 ± 0.62^aB^	57.1 ± 0.85^aA^
ES (%)	0.1	–	88.2 ± 0.81^dD^	84.8 ± 0.70^cC^	82.1 ± 0.69^cB^	79.2 ± 0.79^cA^
	300	3	90.2 ± 0.72^efB^	87.6 ± 0.76^deC^	85.5 ± 0.62^dB^	83.6 ± 0.45^eA^
		6	91.5 ± 0.83^fC^	89.1 ± 0.51^eC^	86.8 ± 0.64^eB^	84.7 ± 0.87^eA^
		9	91.8 ± 0.53^fC^	89.0 ± 0.95^eC^	87.1 ± 0.61^eB^	84.4 ± 0.90^eA^
	400	3	93.4 ± 0.70^gC^	91.1 ± 0.67^fC^	89.3 ± 0.47^fB^	87.1 ± 0.96^fA^
		6	94.6 ± 0.85^ghD^	92.6 ± 0.70^fgC^	90.2 ± 0.44^fgB^	88.0 ± 0.40^fgA^
		9	96.1 ± 0.95^hD^	93.7 ± 0.72^gC^	90.9 ± 0.98^gB^	88.7 ± 0.56^gA^
	500	3	91.2 ± 0.60^fD^	89.2 ± 0.87^eC^	87.1 ± 0.81^eB^	84.2 ± 0.62^eA^
		6	88.9 ± 0.89^deD^	87.0 ± 0.81^dC^	85.0 ± 0.78^dB^	82.6 ± 0.76^dA^
		9	86.6 ± 0.62^cD^	84.6 ± 0.62^cC^	82.9 ± 0.92^cB^	80.7 ± 0.91^cA^
	600	3	87.2 ± 0.91^cdD^	85.3 ± 0.68^cC^	82.6 ± 0.68^cB^	80.9 ± 0.95^cA^
		6	84.1 ± 0.95^bD^	82.1 ± 0.85^bC^	80.1 ± 0.57^bB^	77.8 ± 0.90^bA^
		9	81.5 ± 0.59^aD^	79.4 ± 0.92^aC^	77.4 ± 0.82^aB^	75.5 ± 0.83^aA^

EC, Emulsifying capacity; ES, Emulsifying Stability. Means with different capital letters in the same row indicate significant differences among protein concentrations (*p* < 0.05). Means with different lowercase letters in the same column indicate significant differences among treatments at each pressure level (*p* < 0.05).

The EC and ES of FBPI were strongly dependent on the structural modifications induced by HHP, particularly the balance between protein flexibility and aggregation. As shown in Table 4, both EC and ES increased significantly with pressure up to 400 MPa, reaching maximum values at this level across all concentrations. For instance, at 5% concentration, EC increased from 77.8% (300 MPa, 3 min) to 86.5% (400 MPa, 9 min), while ES rose from 90.2% to 96.1% under the same conditions. This improvement suggests that moderate pressure enhances interfacial functionality by promoting partial unfolding of globular proteins, leading to increased conformational adaptability and improved adsorption at the oil-water interface.

The enhancement of emulsifying properties observed at moderate pressures aligns with the structural modifications identified through FTIR analysis and SH group measurements. These alterations are likely to have improved the capacity of FBPI molecules to adsorb and reorganize at the oil-water interface, promoting the formation of cohesive and viscoelastic interfacial films that enhance emulsion stability and mitigate droplet coalescence (13, 16).

In contrast, pressures exceeding 400 MPa consistently resulted in a decline in both EC and ES. At 5%, EC decreased to 66.8% and ES to 81.5% at 600 MPa (9 min), signifying a deterioration in emulsifying performance. This trend indicates a shift toward aggregation-dominated behavior, whereby excessive unfolding facilitates intermolecular interactions such as disulfide bond formation and β-sheet aggregation. These structural modifications were supported by FTIR spectra, which displayed an increase in aggregate-associated bands, and by the reduction in free SH content at higher pressures. The resulting protein conformations demonstrate reduced solubility, limited diffusivity, and lower surface activity, thereby impairing their capacity to form and stabilize interfacial films (48, 50).

Holding time exerted a secondary but consistent influence. At 300 MPa, increasing treatment time from 3 to 9 min slightly improved EC and ES, indicating progressive structural rearrangement and enhanced interfacial performance. However, at pressures of ≥400 MPa, prolonged treatment resulted in a gradual decrease in emulsifying properties, suggesting that extended exposure favors the reorganization of unfolded proteins into more compact and less functional aggregates. This underscores the importance of regulating both pressure intensity and residence time to maintain optimal functionality.

Protein concentration also played a crucial role. Both EC and ES decreased as concentration increased from 5% to 20%. At 400 MPa and 9 min, EC decreased from 86.5% (5%) to 74.7% (20%), while ES decreased from 96.1% to 88.7%. This behavior indicates that more concentrated systems restrict the ability of proteins to rearrange and adsorb efficiently at the interface. In these conditions, closer intermolecular proximity promotes the formation of compact structures that limit interfacial coverage and reduce film-forming capacity. Consistently, FTIR and SH results demonstrated that structural modifications were less pronounced at higher concentrations, suggesting a reduced responsiveness to pressure-induced conformational changes.

Taken together, the emulsifying behavior of FBPI under HHP reflects a delicate interplay between structural flexibility and aggregation. Moderate pressures (~400 MPa) promote an optimal conformational state that enhances interfacial activity, while higher pressures, longer treatment times, and increased protein concentrations favor the formation of structurally constrained aggregates with reduced functionality. These findings emphasize the necessity to customize HHP conditions to attain improved techno-functional performance in plant-based protein systems, particularly in applications requiring stable emulsions.

#### 3.4.3 Water holding capacity (WHC)

The results of WHC of FBPI as affected by HHP are presented in Table 5. The WHC of FBPI showed a clear dependence on pressure intensity and protein concentration, reflecting structural transitions induced by HHP processing. The increase in WHC observed at moderate pressures (300–400 MPa) can be attributed to partial unfolding of globular storage proteins, which enhances the exposure of polar and charged groups. This structural rearrangement increases protein-water interactions and promotes the formation of a more open and hydrated network capable of retaining water within the matrix. As shown in Table 5, WHC reached its maximum at 400 MPa and 6 min for all concentrations (e.g., 2.53 g/g at 5% and 2.03 g/g at 20%), indicating that optimal hydration is achieved within a defined processing window.

**Table 5:** Water holding capacity (WHC) of fava bean protein isolate (FBPI) samples subjected to high hydrostatic pressure (HHP).

Pressure (MPa)	Time (min)	5%	10%	15%	20%
0.1	–	2.02 ± 0.09^abD^	1.88 ± 0.06^abC^	1.75 ± 0.06^abB^	1.62 ± 0.05^abA^
300	3	2.17 ± 0.07^cdD^	2.02 ± 0.05^cdC^	1.88 ± 0.08^cdeB^	1.74 ± 0.04^cdeA^
	6	2.23 ± 0.04^bcD^	2.08 ± 0.06^deC^	1.94 ± 0.03^efgB^	1.80 ± 0.06^efA^
	9	2.28 ± 0.06^efD^	2.13 ± 0.04^efC^	1.99 ± 0.08^fgB^	1.85 ± 0.05^fghA^
400	3	2.41 ± 0.05^ghD^	2.24 ± 0.04^ghC^	2.09 ± 0.04^hiB^	1.94 ± 0.05^hiA^
	6	2.53 ± 0.07^iD^	2.35 ± 0.06^iC^	2.19 ± 0.04^jB^	2.03 ± 0.07^jA^
	9	2.47 ± 0.07^hiD^	2.29 ± 0.06^hiC^	2.13 ± 0.05^ijB^	1.97 ± 0.05^ijA^
500	3	2.34 ± 0.06^fgD^	2.18 ± 0.07^fgC^	2.03 ± 0.05^ghB^	1.89 ± 0.07^ghiA^
	6	2.25 ± 0.04^defD^	2.10 ± 0.07^defC^	1.96 ± 0.06^efgB^	1.83 ± 0.05^fgA^
	9	2.18 ± 0.03^defD^	2.04 ± 0.06^cdeC^	1.90 ± 0.05^defB^	1.77 ± 0.05^defA^
600	3	2.10 ± 0.05^bcD^	1.96 ± 0.06^bcC^	1.83 ± 0.06^bcdB^	1.71 ± 0.08^bcdA^
	6	2.05 ± 0.06^abD^	1.91 ± 0.07^abC^	1.78 ± 0.06^abcB^	1.67 ± 0.05^abcA^
	9	1.98 ± 0.05^aD^	1.85 ± 0.05^aC^	1.73 ± 0.05^aB^	1.61 ± 0.04^aA^

Means with different capital letters in the same row indicate significant differences among protein concentrations (*p* < 0.05). Means with different lowercase letters in the same column indicate significant differences among treatments at each pressure level (*p* < 0.05).

This improvement is supported by the structural evidence obtained from FTIR and SH analyses. Moderate pressure treatments led to subtle changes in secondary structure without extensive loss of ordered conformations, while the increase in SH groups suggests the exposure of reactive sites that enhance protein-water affinity. These combined effects favor the development of a flexible network with increased hydration capacity, consistent with previous studies reporting enhanced WHC in pulse proteins subjected to moderate HHP (51).

In contrast, the reduction in WHC observed at pressures above 400 MPa indicates a transition toward aggregation-dominated behavior. At 500–600 MPa, WHC decreased progressively across all concentrations, reaching values close to or even approaching those of the untreated samples (e.g., 1.98 g/g at 5% and 1.61 g/g at 20% at 600 MPa, 9 min). This decline suggests that excessive structural disruption promotes the formation of dense protein assemblies with reduced capacity to interact with water. FTIR results revealed an increase in β-sheet structures associated with aggregation, while the decrease in free SH groups indicates the involvement of disulfide bond formation, both contributing to a more compact and less hydrated matrix (42). These structural features reduce matrix porosity and limit the availability of water-binding sites, ultimately impairing water retention.

Treatment time also influenced WHC, although its effect was dependent on pressure level. At 300 MPa, increasing holding time from 3 to 9 min resulted in a gradual improvement in WHC, suggesting progressive structural adaptation. However, at 400 MPa, WHC peaked at 6 min and slightly decreased at 9 min, indicating that prolonged exposure may initiate early stages of structural reorganization toward less hydrated states. At higher pressures, longer treatment times consistently reduced WHC, reinforcing the idea that extended exposure accelerates aggregation and structural compaction.

Protein concentration exerted a strong influence on WHC, with values consistently decreasing from 5% to 20% under all conditions. This trend indicates that more concentrated systems retain less water per unit mass, likely due to reduced free space within the protein matrix and increased intermolecular interactions. In these systems, proteins are less able to reorganize under pressure, resulting in a more constrained structure with limited responsiveness to HHP. This is supported by the FTIR data, where higher concentrations showed smaller variations in secondary structure across pressure levels, suggesting restricted conformational mobility. As a result, the relative improvement in WHC induced by HHP was more pronounced in dilute systems, whereas concentrated matrices exhibited a more limited functional response.

From an application perspective, these findings indicate that moderate HHP treatments can be effectively used to enhance hydration properties in formulations where water retention is critical, such as plant-based beverages or soft gels. In contrast, for high-protein or dense systems, careful control of processing conditions is required to avoid excessive aggregation and loss of functional performance.

#### 3.4.4 Foaming properties

The foaming capacity (FC) and foam stability (FS) of FBPI subjected to HHP are presented in Table 6. Foaming parameters were markedly influenced by pressure level, treatment time, and protein concentration, demonstrating a clearly defined trend characterized by enhancement at moderate pressures followed by a decline at elevated intensities. The foaming capacity (FC) and foam stability (FS) of FBPI were significantly affected by the structural modifications induced by HHP, particularly those impacting interfacial dynamics at the air-water boundary. As shown in Table 6, both FC and FS exhibited notable increases at moderate pressures, attaining their peak values at 400 MPa across all concentrations. For instance, at 5%, FC rose from 30.8% in the untreated sample to 46.1% at 400 MPa (6 min), while FS improved from 81.6% to 91.2% under identical conditions. This enhancement signifies an improved capacity of the proteins to rapidly adsorb, unfold, and reorganize at the interface, thereby facilitating air incorporation and the formation of stable foam structures.

**Table 6:** Foaming properties of fava bean protein isolate (FBPI) samples subjected to high hydrostatic pressure (HHP).

Parameter	Pressure (MPa)	Time (min)	5%	10%	15%	20%
**FC (%)**	0.1	–	30.8 ± 0.79^aD^	28.6 ± 0.75^aC^	26.4 ± 0.44^aB^	24.5 ± 0.49^aA^
	300	3	35.2 ± 0.64^dD^	32.8 ± 0.85^cC^	30.4 ± 0.71^dB^	27.9 ± 0.62^cA^
		6	37.4 ± 0.50^eD^	34.6 ± 0.47^dC^	31.9 ± 0.64^eB^	29.2 ± 0.55^deA^
		9	38.6 ± 0.32^fD^	35.8 ± 0.46^eC^	33.1 ± 0.52^fB^	30.3 ± 0.53^fA^
	400	3	43.5 ± 0.49^hD^	40.2 ± 0.61^gC^	37.0 ± 0.40^hB^	33.8 ± 0.40^hA^
		6	46.1 ± 0.35^jD^	42.8 ± 0.66^iC^	39.5 ± 0.49^jB^	36.2 ± 0.61^jA^
		9	44.8 ± 0.66^iD^	41.5 ± 0.35^hC^	38.2 ± 0.55^iB^	35.0 ± 0.56^iA^
	500	3	40.9 ± 0.59^gD^	37.7 ± 0.57^fC^	34.8 ± 0.46^gB^	31.7 ± 0.47^gA^
		6	38.7 ± 0.42^fD^	35.6 ± 0.64^eC^	32.9 ± 0.61^fB^	30.1 ± 0.38^efA^
		9	36.8 ± 0.36^eD^	33.8 ± 0.56^dC^	31.1 ± 0.55^deB^	28.5 ± 0.32^cdA^
	600	3	33.9 ± 0.38^cD^	31.4 ± 0.50^bC^	29.0 ± 0.36^cB^	26.8 ± 0.59^bA^
		6	31.9 ± 0.72^bD^	29.6 ± 0.57^aC^	27.4 ± 0.35^bB^	25.3 ± 0.59^aA^
		9	31.0 ± 0.74^abD^	28.9 ± 0.57^aC^	26.7 ± 0.55^abB^	24.7 ± 0.38^aA^
**FS (%)**	0.1	–	81.6 ± 0.60^aD^	79.3 ± 0.36^aC^	76.9 ± 0.53^aB^	74.8 ± 0.85^abA^
	300	3	84.0 ± 0.64^dD^	81.5 ± 0.67^dC^	78.9 ± 0.36^cB^	76.6 ± 0.50^deA^
		6	85.4 ± 0.31^eD^	82.7 ± 0.71^eC^	80.0 ± 0.67^dB^	77.7 ± 0.47^fA^
		9	86.1 ± 0.50^eD^	83.5 ± 0.55^efC^	80.8 ± 0.55^deB^	78.4 ± 0.49^fgA^
	400	3	88.9 ± 0.40^gD^	86.0 ± 0.66^gC^	83.1 ± 0.62^fB^	80.4 ± 0.67^hA^
		6	91.2 ± 0.56^iD^	88.1 ± 0.38^hC^	85.1 ± 0.74^gB^	82.2 ± 0.70^iA^
		9	90.0 ± 0.95^hD^	86.9 ± 0.60^gC^	83.9 ± 0.62^fB^	81.0 ± 0.59^hA^
	500	3	87.1 ± 0.38^fD^	84.3 ± 0.50^fC^	81.6 ± 0.74^eB^	79.0 ± 0.44^gA^
		6	85.4 ± 0.70^eD^	82.8 ± 0.78^eC^	80.1 ± 0.62^cB^	77.5 ± 0.59^efA^
		9	83.8 ± 0.46^cdD^	81.2 ± 0.61^cdC^	78.7 ± 0.35^dB^	76.2 ± 0.56^cdA^
	600	3	82.9 ± 0.47^bcD^	80.5 ± 0.59^bcC^	78.0 ± 0.53^bcB^	75.7 ± 0.36^bcA^
		6	82.1 ± 0.35^abD^	79.7 ± 0.47^abC^	77.2 ± 0.46^abB^	75.0 ± 0.38^abA^
		9	81.4 ± 0.50^aD^	79.0 ± 0.46^aC^	76.6 ± 0.44^aB^	74.4 ± 0.31^aA^

Means with different capital letters in the same row indicate significant differences among protein concentrations (*p* < 0.05). Means with different lowercase letters in the same column indicate significant differences among treatments at each pressure level (*p* < 0.05).

This behavior may be attributed to pressure-induced modifications that enhance interfacial activity without causing extensive disruption to the protein backbone. Evidence from SH analysis indicates increased exposure of reactive groups at moderate pressures, which could contribute to stronger intermolecular interactions at the interface. Additionally, FTIR results suggest that secondary structures are only partially altered, thereby allowing proteins to maintain sufficient flexibility to form cohesive and elastic interfacial films. Such films are instrumental in stabilizing bubbles by reducing liquid drainage and delaying coalescence, ultimately leading to improved foam stability (26, 27).

At pressures above 400 MPa, both FC and FS exhibited a consistent decline, signifying a deterioration in foaming performance. For example, at 5%, FC decreased to 31.0%, and FS to 81.4% at 600 MPa (9 min), approaching values comparable to those observed in untreated samples. This decrease indicates that excessive pressure facilitates the formation of protein assemblies with limited interfacial functionality. FTIR data demonstrated an increase in ordered aggregate structures, whereas the reduction in free SH groups supports the formation of more stable intermolecular interactions. These structural attributes likely impede rapid adsorption and restrict the proteins' capacity to reorganize at the interface, thereby resulting in weaker and less resilient foam films (42, 52).

The treatment duration exhibited a pressure-dependent influence on the foaming characteristics. At 300 MPa, elongating the holding time from 3 to 9 min led to a progressive enhancement of both FC and FS, implying that structural modifications that improve interfacial activity necessitate adequate exposure time. Conversely, at 400 MPa, the peak values were generally recorded at 6 min, with marginal declines observed at longer times, indicating the commencement of structural reorganization toward less functional conformations. At elevated pressures, extended treatment intervals consistently reduced foaming performance, reinforcing the notion that prolonged exposure facilitates the formation of less dynamic protein structures.

Protein concentration significantly influences both foaming capacity (FC) and foaming stability (FS), with observed values decreasing progressively from 5% to 20% across all processing conditions. Specifically, at 400 MPa and 6 min, FC decreased from 46.1% at 5% concentration to 36.2% at 20%, whereas FS decreased from 91.2% to 82.2%. This pattern indicates that more concentrated systems hinder the ability of proteins to effectively migrate and disperse at the air-water interface. Under these conditions, enhanced intermolecular interactions facilitate the formation of more compact structures, reducing interfacial coverage and compromising foam stability. Additionally, FTIR analyses revealed minimal structural alterations at higher protein concentrations, implying a restricted response to pressure-induced modifications.

Taken together, the foaming behavior of FBPI under HHP highlights the significance of attaining a balance between structural flexibility and intermolecular interactions. Moderate pressures improve interfacial functionality and foam-forming capacity, whereas higher pressures, extended treatment times, and elevated protein concentrations systematically reduce foaming efficiency by restricting protein mobility and interfacial reorganization. These findings offer valuable insights for customizing FBPI functionality within aerated food systems, where both swift adsorption and film stability are essential.

## 4 Conclusions

This study demonstrates that the effects of HHP on FBPI are strongly dependent on pressure level, holding time, and protein concentration. Moderate pressures (300–400 MPa) promoted partial protein unfolding, particularly at lower concentrations (5–10% w/v), increasing the exposure of reactive groups and enzymatic cleavage sites, which resulted in enhanced *in vitro* digestibility and improved techno-functional properties, including solubility, EC, foaming properties, and WHC. These improvements were supported by FTIR and SH group analyses, which revealed controlled conformational changes characterized by disruption of tertiary structure while largely preserving secondary structure, leading to increased molecular flexibility and interfacial activity. In contrast, higher pressures (≥500 MPa) and longer treatment times induced protein aggregation, as evidenced by increased β-sheet structures and decreased SH content, indicating the formation of intermolecular interactions such as disulfide bonds, which reduced protein solubility, enzymatic accessibility, and functional performance. Additionally, increasing protein concentration (15–20% w/v) limited the extent of pressure-induced structural modifications due to limited molecular mobility, resulting in reduced responsiveness to HHP and lower digestibility and functionality. These findings highlight a biphasic pressure-dependent behavior in FBPI, where moderate processing conditions enhance structural flexibility and functionality, whereas excessive pressure, prolonged treatment, and higher concentrations promote aggregation and functional decline, emphasizing the importance of optimizing HHP conditions according to system composition for the development of high-performance plant-based protein ingredients.

Future research may focus on evaluating the performance of HHP-treated FBPI in food formulations and across different storage conditions. Further investigation into the relationship between pressure-induced structural modifications and protein bioaccessibility will broaden the understanding of HHP applications and support the development of innovative plant-based food.

## Data Availability

The raw data supporting the conclusions of this article will be made available by the authors, without undue reservation.
